# Beyond health care providers’ recommendations: understanding influences on infant feeding choices of women with HIV in the Eastern Cape, South Africa

**DOI:** 10.1186/s13006-019-0201-5

**Published:** 2019-01-31

**Authors:** Oladele Vincent Adeniyi, Anthony Idowu Ajayi, Moshood Issah, Eyitayo Omolara Owolabi, Daniel Ter Goon, Gordana Avramovic, John Lambert

**Affiliations:** 1 Department of Family Medicine & Rural Health, Faculty of Health Science, Walter Sisulu University, Mthatha/East London Hospital Complex, Cecilia Makiwane Hospital, East London, South Africa; 2 Department of Sociology, Faculty of Social Sciences & Humanities, University of Fort Hare, East London, South Africa; 3 Department of Nursing Sciences, Faculty of Health Sciences, University of Fort Hare, East London, South Africa; 4 Department of Infectious Diseases, Medicine and Sexual Health, Mater, Rotunda and University College, Dublin, Ireland

**Keywords:** Exclusive breastfeeding, Infant feeding practice, infant formula feeding, HIV-infected peripartum women, South Africa, WHO guideline

## Abstract

**Background:**

Despite the array of studies on infant feeding practices of HIV-infected women, gaps still exist in the understanding of the underlying reasons for their infant feeding choices. Potential for behavioural change exists, especially in the light of the 2016 updated World Health Organization guideline on HIV and infant feeding. The aim of this paper is to determine the rate of adoption of exclusive breastfeeding in this cohort, examine the determinants of infant feeding choices of HIV-infected women and assess the underlying reasons for these choices.

**Methods:**

This was a mixed methods study conducted between September 2015 and May 2016. It analyses the quantitative and qualitative data of 1662 peripartum women enrolled in the East London Prospective Cohort Study across three large maternity services in the Eastern Cape. Women with HIV reported their preferred choices of infant feeding. In addition, participants explained the underlying reasons for their choices. Descriptive and inferential statistics summarised the quantitative data, while thematic content analysis was performed on qualitative data.

**Results:**

Of the 1662 women with complete responses, 80.3% opted to exclusively breastfeed their babies. In the adjusted model, up to grade 12 education level (AOR: 1.81; 95% CI: 1.14, 2.86), rural/peri-urban residence (AOR:1.44; 95% CI: 1.05, 1.96), alcohol use (AOR: 1.65; 95% CI: 1.25, 2.18), negative or unknown HIV status at booking (AOR:1.85; 95% CI:1.27, 2.70), currently married (AOR:1.43; 95% CI:1.01, 2.02) and WHO Clinical Stage 2–4 (AOR:1.77; 95% CI: 1.15, 2.72) were significantly associated with the decision to exclusively breastfeed. Health care providers’ recommendations, perceived benefits of breastfeeding, unaffordability of formula feeding, and coercion were the underlying reasons for wanting to breastfeed; while work/school-related demands, breast-related issues, and fear of infecting the baby influenced their decision to formula feed.

**Conclusion:**

The majority of HIV-infected women chose to breastfeed their babies in the Eastern Cape. Following up on these women to ensure they breastfeed exclusively, while also addressing their possible concerns, could be an important policy intervention. Future studies should focus on how early infant feeding decisions change over time, as well as the health outcomes for mother and child.

## Background

Globally, an estimated 180,000 children were newly infected with HIV in 2017 [1]. About 90% of these infected children acquired the HIV virus from their mothers during pregnancy and delivery and to a lesser extent, through breastfeeding. The majority of these children are likely to die before the age of five because HIV infection progresses more rapidly in infants than in adults [2]. Studies have shown that adherence to antiretroviral therapy (ART) and breastfeeding exclusively from birth onwards [3] have the potential to reduce postpartum vertical transmission of HIV as well as to lower under-five mortality from all causes [4–7]. In contrast, mixed feeding – breastfeeding supplemented with family foods or infant formula or animal milks – is incontrovertibly linked with increased risks of vertical transmission of HIV [8] and higher infant morbidity and mortality [9].

Despite the known benefits of exclusive breastfeeding (EBF), available evidence suggests that the practice of EBF is not widespread in resource-poor settings of sub-Saharan Africa [10–12], and especially in South Africa.

Mixed feeding practice is the most prevalent infant feeding practice for babies younger than 6 months in South Africa [13–17]. Nationally representative studies show that the rate of EBF ranges from 25.7 to 32%, among the lowest in the world [18, 19]. Madiba and Langa [20] argue that to practise exclusive breastfeeding, women may have to go against their cultural norms, which is very difficult, irrespective of a woman’s HIV status.

South Africa is one of the countries with the highest burden of HIV [21]. Owing to the high prevalence of HIV among pregnant women (30.8%) [22, 23], the South African Government has made elimination of mother to child transmission (MTCT) of HIV a national health priority [24]. Prior to 2011, South African infant feeding policy promoted exclusive formula feeding [24]. All public health facilities provided women living with HIV with free infant formula for infants up to 6 months of age [24]. In October 2010, a circular was passed to effectively end the programme by 2012 [24, 25], as South Africa embraced the WHO 2010 guidelines, electing to promote EBF only [24]. South Africa, through the Tshwane Declaration, declared itself as a country that actively promotes, protects and supports exclusive breastfeeding [24]. The Tshwane Declaration is important in that it ended the free formula programme.

In 2016 World Health Organization (WHO) updated its HIV and Infant Feeding Guideline. This recommends that HIV-infected women exclusively breastfeed their infants for the first 6 months after birth, introduce appropriate complementary feeding after 6 months, adhere to ART, and continue breastfeeding for up to 2 years of the baby’s life [26]. Under the WHO option B+ programme adopted in South Africa, every HIV-infected pregnant woman is provided with infant-feeding counselling during antenatal clinic visits [26].

Despite the widespread infant-feeding counselling, available evidence suggests that many HIV-infected women do not follow health care providers’ (HCPs) advice [27–29]. However, studies suggest that infant counselling advice (mostly done by nurses, community health workers and doctors) is very important to mothers in South Africa [29, 30]. The reasons for infant feeding choices made by HIV-infected pregnant women are multifaceted and vary from one woman to another. [17, 27, 31–33]. Cultural norms about infant feeding practice, stigmatization, inadequate counselling, inadequate financial resources, and infant feeding knowledge are among the factors influencing the choice of feeding options for HIV-infected mothers [17, 27, 31–33]. In South Africa, the transition from exclusive formula feeding and provision of free formula milk to promotion of exclusive breastfeeding has created a situation whereby advice from HCPs is often insufficient or contradictory [25, 34]. In other words, health care providers’ advice on exclusive breastfeeding often contradicts lay knowledge and what is advised in the household [3]. While some women would follow health care providers’ advice on infant feeding, others are unable to, due to their peculiar needs [17, 27, 31–33]. HIV-infected women’s knowledge on breastfeeding is not shaped only by HCPs [17, 27, 31–33]. Significant others (parents, sisters, husbands and in-laws) often influence a woman’s knowledge about breastfeeding [4, 30, 31]. These sources of information may conflict at times, but we do not know how this influences the infant feeding practices of women living with HIV. A study indicated that the meanings HIV-infected women ascribed to EBF influenced their understanding of breast milk insufficiency, abrupt weaning and mixed feeding in the context of preventing mother-to-child transmission of HIV [33].

Despite intensified counselling on EBF for HIV-exposed infants at all the health facilities in South Africa, the practice of mixed feeding still persists [28, 29]. Gaps still exist in the understanding of the underlying reasons for infant feeding choices and the potential for behavioural change remains, especially in the light of the 2016 updated WHO Guideline on HIV and infant feeding. The objectives of this paper are to determine the rate of adoption of exclusive breastfeeding post-delivery, examine the determinants of infant feeding choices of HIV-infected women and also, assess the underlying reasons for their choices.

## Methods

### Study design and settings

In this mixed methods study, both the quantitative and qualitative data of 1662 HIV-infected women enrolled in the East London Prospective Cohort Study were extracted. Data collection took place in three large health facilities in the Buffalo City/Amathole districts of the Eastern Cape Province, South Africa, between September 2015 and May 2016. These facilities provide health services for 1,674,637 people living in rural, peri-urban and urban areas in the selected districts. The three facilities represent the three levels of health care in the province: tertiary, regional and district health facilities. Both qualitative and quantitative data were obtained concurrently. The quantitative component involved an interviewer-administered, semi-structured questionnaire. Trained research assistants administered the questionnaire to participants in private offices provided for this purpose by the hospital managements of selected health facilities.

### Participants and procedure

All HIV-infected women who birthed at the maternity centres of the selected hospitals during the study period were recruited consecutively into the study. Participants were excluded if they were either HIV-negative or still pregnant during the study period. Participants were recruited at the post-natal wards of maternity centres within 24 h of vaginal delivery and 72 h for caesarean section delivery. Overall, 1709 eligible participants were recruited into the study. The full study methodology has been published elsewhere [35].

The questionnaire designed for the larger study, which examined outcomes of the prevention of mother-to-child transmission (PMTCT) programme in the Eastern Cape, South Africa, was piloted among 10 pregnant women at one of the study sites to ascertain the validity of the instrument. For the purpose of this study, responses to questions on the chosen infant feeding method and on demographic characteristics were extracted from the database as well as participants’ clinical characteristics. To determine the rate of exclusive breastfeeding, participants were asked: what is the infant feeding option chosen for your baby? The following mutually exclusive options were provided for participants to choose from: exclusive breastfeeding, exclusive formula feeding, mixed feeding and undecided. To understand the underlying reasons for infant feeding choices, we asked all participants an open-ended question on why they chose their infant feeding method. Responses were documented in the questionnaire, and captured separately for content analysis. In order to ensure data validity, we recruited qualified and well-trained research assistants to capture responses of the participants. In addition, the principal investigator and the research team reviewed the responses frequently and provided feedback to the research assistants.

### Statistical analysis

Data were extracted from the electronic database in an Excel format and transferred to the Statistical Package for the Social Sciences, version 24.0 (SPSS, Chicago, IL, USA). Simple frequencies and means were used to summarise categorical variables. Chi-square statistics were used to examine determinants of infant feeding choices. The qualitative data generated through the open-ended question on reasons for the preferred infant feeding methods were recoded manually and categorised into themes using the recursive abstraction method [36]. Descriptive statistics were used to summarise the themes. However, to provide context and in-depth analysis of responses emerging from the qualitative data on reasons for the choice of infant feeding methods, verbatim quotes of participants’ responses were used to describe the themes. Content analysis was used for the data analysis, and it involved four steps. Responses were read for familiarisation and re-read for understanding before coding was performed. Emerging codes were then grouped under themes, which were refined several times. The authors discussed the emerging themes a number of times, and re-categorised the codes under five main themes.

## Results

The mean age of the study participants was 29.63 (SD ± 6.2) years. Few participants were below 21 years (7.1%), married (18.9%), had tertiary education (6.5%), and employed (24.8%) (Table 1). The majority of the participants were non-smokers (89.9%), non-users of alcohol (61.2%), knew their positive status at booking (80.9%), had disclosed their HIV status to a family member (80.0%) and sexual partner (74.4%). Only a third of the participants had a peripartum CD4 count equal or greater than 500 (Table 1).

**Table 1:** Demographic, clinical and behavioural characteristics of respondents

Variables	Frequency (*n* = 1709)	Percentage
Age		
20 and below	121	7.1
21–25	370	21.7
26–30	462	27.0
31–35	434	25.4
36–40	259	15.2
Above 40	63	3.7
Marital status		
Married	312	18.9
Single	1131	68.4
Co-habiting	186	11.3
Divorce/Separated	24	1.5
Type of residence		
Rural	562	33.6
Semi-urban	782	46.8
Urban	327	19.6
Educational level		
No formal education	5	0.3
Grade 1–6	86	5.1
Grade 7–12	1479	88.0
Tertiary	110	6.5
Employment status		
Unemployed	1272	75.2
Employed	420	24.8
Smoking status		
Smoking during pregnancy	92	5.4
Quit smoking during pregnancy	80	4.7
Never smoked	1529	89.9
Alcohol use		
Drank during pregnancy	230	13.5
Stopped during pregnancy	431	25.3
Never drank	1043	61.2
Reported HIV status at booking		
Positive	1356	80.9
Negative	87	5.2
Unknown	233	13.9
PP CD4		
Normal	559	32.7
Mild immunosuppression	359	21.0
Advanced immunosuppression	380	22.2
Severe immunosuppression	411	24.0
Disclosure to family		
No	337	20.0
Yes	1350	80.0
Disclosure to partner		
No	431	25.6
Yes	1253	74.4
Self-reporting		
No	373	22.7
Yes	1270	77.3
Pick up HAART		
No	290	18.1
Yes	1313	81.9
Defaulted ARV		
No	1407	87.8
Yes	196	12.2

### Demographic, behavioural and clinical determinants of infant feeding choices

Of the 1662 women with complete responses, 80.3% opted to exclusively breastfeed their babies. Only seven women were undecided which infant feeding method they would practise. In the chi-square analysis, only education, employment status, alcohol use, and sero-status at booking, were significantly associated with infant feeding choices (Table 2). The results of the adjusted and unadjusted logistics regression models are presented in Table 3. In the unadjusted model, age 30 years and below, up to grade 12 education level, ever drank alcohol, negative or unknown HIV status at booking, and being an individual in WHO Clinical Stage 2–4, were independently associated with the choice to breastfeed exclusively. However, in the adjusted model, only up to grade 12 education level, rural/peri-urban residence, never drank alcohol, negative or unknown HIV status at booking, being married and being an individual in WHO Clinical Stage 2–4, were associated with the decision to exclusively breastfeed.

**Table 2:** Association between demographic, clinical, behavioural characteristics, and infant feeding practices

Variables	Total n (%)	Exclusive Breastfeeding n (%)	Exclusive Formula Feeding n (%)	*p-value*
All	1662 (100%)	1334 (80.3)	321 (19.3)	
Age				
20 and below	118 (7.1)	98 (83.1)	20 (16.9)	0.096
21–25	356 (21.5)	300 (84.3)	56 (15.7)	
26–30	448 (27.1)	363 (81.0)	85 (19.0)	
31–35	422 (25.5)	321 (76.1)	101 (23.9)	
36–40	250 (15.1)	198 (79.2)	52 (20.8)	
Above 40	61 (3.7)	50 (82.0)	11 (18.0)	
Marital status				
Married	306 (19.1)	255 (83.3)	51 (16.7)	0.428
Single	1091 (68.1)	864 (79.2)	227 (20.8)	
Co-habiting	182 (11.4)	147 (80.8)	35 (19.2)	
Divorced	24 (1.5)	20 (83.3)	4 (16.7)	
Type of residence				
Rural	536 (33.1)	429 (80.0)	107 (20.0)	0.129
Semi-urban	763 (47.1)	627 (82.2)	136 (17.8)	
Urban	320 (19.8)	246 (76.9)	74 (23.1)	
Educational level				
Illiterate	5 (0.3)	5 (100.0)	0 (0.0)	0.036
Grade 1–6	74 (4.5)	60 (81.1)	14 (18.9)	
Grade 7–12	1441 (88.5)	1168 (81.1)	273 (18.9)	
Tertiary	108 (6.6)	76 (70.4)	32 (29.6)	
Employment status				
Unemployed	1234 (75.2)	1004 (81.4)	230 (18.6)	0.029
Employed	409 (24.8)	312 (76.8)	94 (23.2)	
Smoking status				
Smoking during pregnancy	91 (5.5)	75 (82.4)	16 (17.6)	0.468
Quit smoking during pregnancy	80 (4.9)	68 (85.0)	12 (15.0)	
Never smoked	1478 (89.6)	1181 (79.9)	297 (20.1)	
Alcohol use				
Drank during pregnancy	220 (13.3)	188 (85.5)	32 (14.5)	< 0.001
Stopped during pregnancy	427 (25.8)	365 (85.0)	64 (15.0)	
Never drank	1005 (60.8)	777 (77.3)	228 (22.7)	
Disclosure to family				
No	333 (20.3)	275 (82.6)	58 (17.4)	0.138
Yes	1308 (79.7)	1043 (79.7)	265 (20.3)	
Disclosure to partner				
No	427 (26.1)	352 (82.4)	75 (17.6)	0.115
Yes	1211 (73.9)	964 (79.6)	247 (20.4)	
Self-reporting of adherence				
No	367 (23.0)	289 (78.7)	78 (21.3)	0.230
Yes	1232 (77.0)	993 (80.7)	238 (19.3)	
Pick up HAART				
No	288 (18.5)	230 (79.9)	58 (20.1.)	0.387
Yes	1270 (81.5)	1026 (80.8)	244 (19.2)	
Defaulted ARV				
No	1369 (87.8)	1103 (80.6)	266 (19.4)	0.173
Yes	190 (12.2)	147 (77.4)	43 (22.6)	
HIV status at booking				
Positive	1310 (80.6)	1028 (78.5)	282 (21.5)	0.002
Negative	87 (5.4)	78 (89.7)	9 (10.3)	
Unknown	228 (14.0)	196 (86.0)	32 (14.0)	
PP CD4				
Normal	552 (33.4)	447 (81.0)	105 (19.0)	0.242
Mild immunosuppression	355 (21.5)	277 (78.0)	78 (22.0)	
Advanced immunosuppression	373 (22.5)	311 (83.4)	62 (16.6)	
Severe immunosuppression	375 (22.7)	295 (78.7)	80 (21.3)	
WHO Clinical stage				
Stage 1	1447 (87.4)	1151 (79.5)	296 (20.5)	0.055
Stage 2	188 (11.4)	159 (84.6)	29 (15.4)	
Stage 3	16 (1.0)	16 (100.0)	0 (0.0)	
Stage 4	4 (0.2)	4 (100.0)	0 (0.0)

**Table 3:** Adjusted and unadjusted models showing determinants of exclusive breastfeeding

Variables	Unadjusted Odds ratio (95%CI)	Adjusted odds ratio (95%CI)
Education level		
No education/ grade 1–12	1.81 (1.17, 2.79)*	1.81 (1.14, 2.86)*
Tertiary education		
Place of residence		
Rural/peri-urban	1.31 (0.97, 1.76)	1.44 (1.05, 1.96)*
Urban		
Alcohol use		
Ever drank alcohol	1.68 (1.30, 2.19)***	1.65 (1.25, 2.18)***
Never drank alcohol		
HIV status at booking		
Negative/unknown	1.83 (1.29, 2.61)*	1.85 (1.27, 2.70)*
Positive		
Age		
30 years and below	1.36 (1.07, 1.74)*	1.20 (0.92, 1.57)
Above 30 years		
WHO Clinical stage		
Stage 2–4	1.59 (1.05, 2.40)*	1.77 (1.15, 2.72)*
Stage 1		
Marital status		
Currently married	1.29 (0.93, 1.79)	1.43 (1.01, 2.02)*
Not currently married		

***represents *p*-value < 0.001; *represents *p*-value < 0.05; *CI* Confidence Interval

### Underlying reasons for infant feeding choices among women infected with HIV

The results of qualitative data are summarised under five main themes (Table 4).

**Table 4:** Themes and subthemes on reasons for infant feeding choices of women with HIV

Themes	Subthemes
Health workers’ recommendations	• Advised by health workers to breastfeed for at least six months. • Advised by nurse that breast milk is the best option for the baby. • Advised by nurse that breast milk is healthy and prevents diseases. • Recommended by doctor to formula feed as there is 25% chance of infection through breastfeeding.
Perceived benefits of breastfeeding	• Best choice. • Good for the baby. • Baby will grow fast. • Unaffordability of formula. • Breast milk is cheap. • Breast milk makes baby strong. • I love to breast milk • Breast milk helps prevent baby from getting becoming sick or getting infections • It is the healthiest and the most nutritious option • Baby will have strong muscle • Previous experience was good • Want to experience breastfeeding for the first time • Breast feeding connects the mother to the baby
Work/school related	• Returning to school • Returning to work • Looking for employment • Farm work is too much and won’t be able to stay with the baby
Fear of transmitting the infection to their babies	• I am scared to breastfeed because the baby will be infected
Breast related issues	• Breast milk not secreting/flowing • Painful breast • Breasts have warts
Other reasons	• Baby refused to suck • Forced • Domestic violence • Wanted to give baby up for adoption • Advised by mum/spouse • No reason

### Health care providers’ recommendation

For many women, the infant feeding choice was as simple as following the instruction of their health workers. Health care providers’ recommendation was the main reason given for the decision to breastfeed, in about half of the women who chose the EBF option. Many women living with HIV were convinced, based on the advice they got from their providers, that breastfeeding and ART adherence would enable them to prevent HIV transmission to their infants.

*“I was told that HIV-positive mothers should breastfeed for six months” (36 year old, on ART since 2014).*

However, he responses of a few women suggested that health care providers might have influenced their choice of formula feeding. Some women reported that doctors and nurses indeed recommended formula feeding, especially for women with high viral load while on Aluvia-based regimen, and those with unknown viral load, as the best method for the prevention of HIV transmission to their infants. When a young mother was asked why she had chosen to formula feed, she stated:

*“My doctor recommended formula feeding, as there is a 25% chance of infection through breastfeeding” (24 year old; started ART during the index pregnancy).*

Another woman stated:

“*My CD4 count and viral load status are unknown. The nurses said that formula feeding is the safer option” (28 year old; initiated ART in 2012).*

One participant stated that she had been recommended by HCPs to formula feed because she recently had an operation on her breast.

### Perceived benefits of breastfeeding

Besides health care providers’ recommendations, perceived benefits of breast milk were among the main reasons why women infected with HIV chose exclusive breastfeeding. This is the second most stated reason for choosing exclusive breastfeeding. Many women appeared knowledgeable about the benefits of breastfeeding, and this knowledge was obtained from the health care providers, friends, mothers and personal research. Among the stated benefits of breast milk were: breast milk prevents diseases, enables the baby to be well-fed, helps the baby to develop muscles, helps baby to grow fast, and it is nutritious. In general, most women were convinced that breastfeeding is the healthiest and the most nutritious option for their infants. When asked to state her reason for her preference of breast-feeding, a 27 year old woman stated thus:

*“Breastfeeding is the best choice, it is the healthiest and the most nutritious option*” (*Initiated ART in 2013*).

Another 25 year old who started ART in year 2015 stated thus;

*“I want the baby to grow and breast milk helps prevent diseases and illness”.*

Many women simply stated that formula feeding was unaffordable and even though some would prefer formula feeding, their choice was unrealistic due to their socioeconomic conditions. Relaying her reason for choosing breastfeeding, a woman stated:

“*What other option do I have? I am unemployed, I cannot afford exclusive formula feeding and government does not provide baby milk*” (35 year old; on ART since 2009).

For a few of the participants, the choice of breastfeeding was predicated on previous positive experiences with the method. Generally, these women described their previous experience with breastfeeding as good. Specifically, their experience of adopting breastfeeding and being able to prevent HIV transmission to their infants in the previous pregnancy motivated them to want to breastfeed in the index pregnancy. In contrast, negative experience with breastfeeding, although reported by few women, motivated some women to prefer formula feeding. This view was captured in the response of a woman who had previously had a baby infected with HIV while mixed feeding:

“*My second child is HIV+. So, I have decided not to breastfeed, as the previous child got infected through mixed feeding as I was not educated about HIV at the time” (32 year old; on ART since 2010).*

### Work/school-related reasons

Many women who chose the formula-feeding option did so because they were either returning to school and work, or searching for work. They appeared convinced that exclusive formula feeding was the most appropriate feeding option for their babies, instead of exclusive breastfeeding. Similarly, many young women who were students opted for formula feeding because they would soon be returning to school, and their mothers would have to care for their infants. To them, formula feeding was a better option than expressing breast milk. In addition, a few women (mostly residents of rural areas) believed they did not have time to breastfeed, because they needed to move to the city to look for jobs.

In general, most of these women (who chose formula feeding due to perceived inability to breastfeed) appeared to have erroneously believed that formula feeding from birth is more appropriate when an individual is unable to breastfeed exclusively for the recommended six months. The rationale for their choice stemmed from their belief that starting to breastfeed, but switching to formula before the recommended six-month exclusive breastfeeding time elapsed would increase the risk of HIV transmission to infants. Nonetheless, a few women in this category stated that, due to their busy schedules, they would exclusively breastfeed their infants for only three months, and subsequently introduce additional food.. This view is captured in the response of a 27-year-old mother who stated:

*“I will switch to formula at 3 months as I will be going back to work.” (on ART since 2011).*

### Fear of transmitting the infection to their babies

Fear of transmitting HIV to their babies was among the most stated reasons for choosing formula feeding. Many women who chose formula feeding did so because they were convinced of it being the best option for their babies. Women who were scared of infecting their babies appeared to favour formula feeding over breast-feeding. These women were convinced that formula feeding is the best feeding option in order to completely eliminate the risk of HIV transmission. A 39-year-old mother, who had been on ART since 2007, said:

*“I am scared to breastfeed because the baby will be infected.” (39 year old; on ART since 2007).*

The fear, in some cases, appears to be informed by their non-adherence to ART. A woman explained that she had defaulted on the use of her medication in the past, and was concerned that she might do so again and infect her baby, should she decide to breastfeed. To her, of utmost priority was preventing the transmission of HIV to her baby, and adopting formula feeding would enable her to ensure this. When asked why she chose formula feeding, she responded:

“*I defaulted on the use of my medication. I am scared of infecting my baby, so, I think formula feeding is the best feeding option in order not to infect my baby.” (35 year old; on ART since 2008).*

Yet some women’s choices of formula feeding were motivated by the reasons of their high viral load and low CD4 counts. A woman stated that: “*my viral load was too high to breastfeed”,* when ask why she had chosen formula feeding. A few women stated that providers advised them not to breastfeed, because their CD4 and viral load results were not yet out. The narrative of a young mother captured this view:

“*I was advised to choose formula because my CD4 and viral load results were not yet out.” (31 year old; on ART since 2012).*

### Breast-related issues

Breast-related issues were stated by many women as a reason for choosing formula feeding. The main breast-related issue stated was the belief that they had insufficient milk. Even though some of these women (whose breasts did not secrete enough milk) would have preferred to breastfeed, having been encouraged to do so, the small amount of breast milk secreted made this option unrealisable without ongoing help to increase their milk supply. Their choice of formula feeding appeared to be the best option in order not to starve their babies. This is corroborated by the response of a 30-year-old mother:

*“I will say, use formula because the breastmilk is not coming out, but nurse had advised me to breastfeed.” (30 year old; on ART since 2013).*

Another woman stated*:* “*I think the baby is not getting enough milk from breastfeeding, so I will use formula feeding.” (26 year old; initiated ART during the index pregnancy).*

In addition, a few women chose formula feeding due to experience of pain in their breasts, while one woman reported the presence of lumps on her breasts.

### Others reasons for infant feeding choices

The baby refused to suck, domestic violence, mother and spouse’s recommendation, placing baby up for adoption, and coercion were other reasons given for infant feeding choices. A few women stated that their choice of breastfeeding was due to the advice they had received from their significant others (mother, sister, aunty, and elders). Although mentioned by only two women in the cohort, coercion ranked among the reasons for choosing to breastfeed. When asked who forced them to breastfeed, mothers and partners were mentioned. A few women who had never breastfed chose breastfeeding just to experience how it feels. Similarly, a few who used formula feeding for their previous babies chose breastfeeding because they wanted to experience it. For these categories of women, their choices appeared to be centred on their belief that breastfeeding helps to connect mothers to their babies.

## Discussion

The objectives of this paper were to determine the rate of adoption of exclusive breastfeeding post-delivery, examine the determinants of infant feeding choices of HIV-infected women and assess the underlying reasons for their choices. The promotion of exclusive breastfeeding was adopted in South Africa from January 2011 [24, 25]. We found that the majority of women opted for exclusive breastfeeding. This is not surprising considering that exclusive breastfeeding is the infant feeding method recommended during prenatal and postnatal counselling of women living with HIV in the study settings [24]. The 2016 WHO updated Guideline on HIV and infant feeding states that women living with HIV (including those whose infants are HIV uninfected or of unknown sero-status) should exclusively breastfeed their infants for the first six months of life, then continue to breastfeed until the baby turns two years old while adding supplementary feeding methods [26]. Exclusive breastfeeding combined with adherence to ART is safe; thus, all parturient women living with HIV are counselled at all antenatal clinics in South Africa on the benefits of this feeding choice. Our finding that most women chose exclusive breastfeeding is consistent with a study conducted in Mpumalanga, South Africa [30].

Our analysis shows that the primary reasons for choosing exclusive breastfeeding were the recommendation of the attending health care provider and the perceived benefits of exclusive breastfeeding. Health care providers often recommend exclusive breastfeeding for women with HIV by stressing its advantages; and most women simply comply with their recommendations. Counselling on infant feeding options is one of the core issues of the PMTCT programme and is discussed at antenatal clinics across the country [29, 37]. Thus, all pregnant women living with HIV are counselled on the appropriate infant feeding methods [29, 37]. Nonetheless, our study shows that women are influenced by other factors beside the health care providers’ recommendations.

It became evident in the semi-structured interviews that most women living with HIV appear to clearly understand the benefits of exclusive breastfeeding, although possibly not aware of the many separate risks of infant formula feeding. They generally consider breast milk to contain nutrients that will aid the growth and development of their infants. Some other women consider breast milk to be safe and healthy and are certain that breast milk will help protect their infants from infections and diseases. In general, most women were convinced that breastfeeding is the healthiest and the most nutritious option for their infants. Their knowledge of the benefits of exclusive breastfeeding did not only emanate from health care providers, but also from their parents, Internet, friends, relatives, and prior experiences. There is an array of literature indicating that breast milk does not only provide nutrients needed by infants in their first six months of life to protect them against common childhood diseases, but also may have longer-term benefits such as cutting the risk of overweight and obesity in childhood and adolescence [9, 12, 38, 39].

Beyond health care providers’ recommendations and consistent with previous literature [30, 32], women generally consider breastfeeding to be cheap, and formula feeding to be very expensive. Even though some women would have preferred formula feeding for other reasons, their choices were unrealistic due to their socioeconomic statuses. Irrespective of the perceived benefits of formula feeding, the high rate of unemployment and poor socioeconomic status of the study population made this feeding option unrealistic for the majority of the women. In addition, positive experience with breastfeeding with previous infants appeared to reinforce the beliefs of previously infected multiparous women about the benefits of breastfeeding, while some others simply wanted to experience breastfeeding.

Even though our finding showed that most women living with HIV chose exclusive breastfeeding, their choice of exclusive breastfeeding post-delivery did not guarantee that all these women would eventually practise exclusive breastfeeding for six months. In fact, available evidence indicates that mixed-feeding is the most prevalent infant feeding method in South Africa [14–16] and in developing countries in general [10]. Nonetheless, the decision to exclusively breastfeed shown by the majority of the women is an important step. Following up on these women with clinical and practical support to ensure they breastfeed exclusively, while also addressing their possible concerns, could be an important policy intervention. A study shows that intervention delivery in a combination of settings (concurrently at home and community, health systems and community, health systems and home settings) seemed to create higher improvements in breastfeeding rates [40]. A follow-up study is already commissioned to track how many of these women completed the recommended six-month exclusive breastfeeding and the total duration of breastfeeding.

Our study also shows that despite the infant feeding counselling and education about the benefits of breastfeeding, many women still favour exclusive formula feeding over EBF. We found that about one in five women intended to exclusively formula feed. This proportion of women favouring exclusive formula feeding is staggering and suggests an urgent need for re-educating women living with HIV not only on the benefits of breastfeeding, but also the separate detrimental impacts of formula feeding. It is well established that breastfeeding is nutritionally and immunologically superior to formula feeding [10, 41]. This important information should be unequivocally communicated to all women at every antenatal and postnatal care visit.

The South Africa infant feeding guidelines [42] still have some grey areas regarding the appropriate feeding choices of mothers who would be with their babies only for a 3-month post-delivery period. Owing to the lack of clarity on this issue, women are left to make a choice of whether to formula feed or breastfeed for the 3 month duration they will be with their babies. Wealth care providers still recommend both formula and breastfeeding depending on the circumstances of women.

Our analysis reveals that age over 30 years, urban residence, tertiary education and prior HIV diagnosis were associated with the decision not to breastfeed. One plausible explanation why older women are more likely not to exclusively breastfeed is that they might have given birth to at least one child between 2000 and 2011, when exclusive formula feeding was the favoured policy in South Africa [34]. Thus, it is plausible that some of them still have challenges reconciling the contrasting information on infant feeding practice.

Consistent with a previous study [30], disclosure of HIV status, non-adherence to ART, CD4 counts and postpartum viral load were not associated with the choice of formula feeding. However, in the semi-structured interviews, the underlying reason for the decision to formula feed was work and school demands. Our findings corroborate a previous study that indicates that women’s work and employment conditions hinder their ability to breastfeed [41]. There is an array of evidence showing that favourable work place policy is associated with an increase in exclusive breastfeeding [43, 44]. Improving workplace policy in the study setting could be an important policy consideration in order to promote exclusive breastfeeding.

This study found a significant association between tertiary education and exclusive formula feeding. The plausible explanation for this finding is that young mothers desire to return to school after child delivery. This explanation is supported by our semi-structured interview, which found that women wanting to return to school is among the main reasons for not intending to breastfeed. Generally, the perspective of mothers on why breastfeeding is inappropriate suggests a lack of knowledge about the WHO updated guideline on breastfeeding. Even though many mothers wanted to return to work and school, a short duration of breastfeeding would have been far more beneficial compared to no breastfeeding at all [26].

In addition, a few women still do not want to breastfeed due to their fear of transmitting HIV to their infants. This finding is consistent with previous studies [5, 45–48]. Overall, our data suggests that the fear of transmitting HIV to infants has been largely allayed. Nonetheless, the fear still exists for some women, and probably explains why women who reported HIV positive prior to the index pregnancy were twice as likely to prefer not to breast feed. This fear is driven to some extent by any previous negative experience with breastfeeding.

Lastly, a few women chose formula feeding due to breast-related issues, a finding consistent with a Chinese study [45]. The most frequently stated breast-related issue is insufficient breast milk. These women worry about starving their babies were they to choose exclusive breastfeeding. They preferred exclusive formula feeding to a mixed feeding approach, which they mostly considered to be unsafe [3].

### Strength and limitations

The findings of this study should be interpreted within the context of its limitations. The cross-sectional nature of the data indicates that any association reported could not be interpreted as causation. The decision to breastfeed exclusively does not equate to the practice of exclusive breastfeeding for the recommended six months. A follow-up study is needed to track the completion rate of the six-month recommended duration of exclusive breastfeeding. Nonetheless, the large dataset and the triangulation of quantitative and qualitative data is an important strength of this study.

## Conclusion

Feeding choice is motivated by a host of very important reasons, as well as the health care providers’ recommendations. The main reasons for choosing formula feeding emerged as fear of breastmilk HIV transmission, work and school demands, and knowledge gaps about both breastfeeding and infant formula. The majority of mothers of HIV-exposed infants preferred exclusive breastfeeding. Following up on these women to ensure they breastfeed exclusively, while also addressing their possible concerns, would be an important policy intervention. Future studies should focus on how early infant feeding decisions change over time, and the outcomes for both maternal and child health.

## Abbreviations

ART: Antiretroviral therapy
EBF: Exclusive breastfeeding
EFF: Exclusive formula feeding
ERF: Exclusive replacement feeding
HIV: Human immunodeficiency syndrome
MTCT: Mother-to-child- transmission of HIV
UNICEF: United Nation Children’s Fund
WHO: World Health Organization
